# Autism Spectrum Disorder and Suicide: A Case Report

**DOI:** 10.7759/cureus.64451

**Published:** 2024-07-13

**Authors:** Abrar N Fatani, Nouf M Alasiri, Osama A Gasem

**Affiliations:** 1 Department of Psychiatry, Mental Health Hospital, Jeddah, SAU; 2 Department of Pediatrics, King Abdulaziz Hospital, Jeddah, SAU

**Keywords:** autism spectrum disorder, diagnosis, attention-deficit/hyperactivity disorder (adhd), self-injurious behavior, suicide attempt

## Abstract

Autism spectrum disorder (ASD) and attention deficit hyperactivity disorder (ADHD) significantly impact mental health, increasing the risk of severe behaviors, including suicidality. This case report examines a 13-year-old boy with ASD and ADHD who presented to the emergency department with homicidal and suicidal ideations. Despite being prescribed risperidone and carbamazepine, his noncompliance with medication and therapy, combined with significant social stressors like physical abuse by his stepfather and his mother’s mental health issues, exacerbated his condition. His aggressive actions toward siblings and self-harm attempts highlight the severe behavioral manifestations of these conditions. The case underscores the necessity for comprehensive and consistent intervention strategies, robust support systems, and regular follow-ups to manage ASD and ADHD effectively and mitigate the risk of severe outcomes.

## Introduction

Autism spectrum disorder (ASD) is a group of complex developmental conditions that involves persistent challenges in social communication, restricted interests, and repetitive behaviors. The degree of impairment varies among individuals, impacting their daily functioning and quality of life to different extents [1]. ASD affects approximately 2.3% of children aged 8 years and about 2.2% of adults in the United States [2]. Comorbid conditions like attention deficit hyperactivity disorder (ADHD) frequently accompany ASD, complicating clinical assessment and potentially exacerbating behavioral and psychiatric challenges [3]. People with ASD face heightened risks of suicidal behaviors, encompassing thoughts, attempts, and completed suicides, compared to the broader population [4]. Social and communication impairments have also been identified as risk factors for suicide attempts ​[5]. In this case report, we present a 13-year-old boy with a long-standing diagnosis of ASD and ADHD who exhibited severe suicidal and homicidal ideations and behaviors. His history includes multiple episodes of aggression, suicidal attempts, and significant behavioral issues compounded by noncompliance with medication and therapy due to family neglect.

## Case presentation

A 13-year-old boy presented to the emergency department (ED) with his mother, complaining of homicidal and suicidal thoughts. According to his mother, he had repeatedly hit his brother's head against the ground, and they were unable to restrain him until she intervened by hitting his head against the wall. He also bit his other brother on the leg and arm, inflicting severe wounds. The patient expressed a desire to die and attempted to jump from a second-floor window, but his mother prevented him and brought him to the ED. In her own words, the mother stated, “I don’t want the child anymore. Let the government handle him.”

The patient has been diagnosed with ASD and ADHD for the past 10 years and is currently taking risperidone and carbamazepine. According to his mother, the patient’s birth and developmental history are normal except for some speech delay. He has no contact with his biological father and has been physically abused by his stepfather. At the age of 8, he was transferred to an autism center but did not follow up regularly and was noncompliant with his medication. He has a history of visiting multiple doctors at various centers over the years. Four years ago, he experienced an episode of depression and anxiety, which escalated to suicidal and homicidal thoughts and gestures. At that time, he was started on escitalopram, and his suicidal and homicidal symptoms were well controlled. Two years ago, he again began having suicidal thoughts and expressed a desire to harm his younger siblings. His medications, risperidone and carbamazepine, were increased, but due to his mother’s negligence, he remained noncompliant with therapy sessions.

Over the past three months before his ED admission, the patient has had difficulty sleeping and has exhibited suicidal and homicidal thoughts and actions, including attempting to strangle himself, trying to jump out of a window, grabbing a kitchen knife to kill his brother, and experiencing visual hallucinations. The patient used to attend a private school for children with ASD but dropped out this year due to aggressive behavior toward his teachers, though he never hit other students. His mother also exhibits symptoms of anxiety and depression and faces social stressors, such as conflicts with her second husband. She isolates the patient from his brothers, fearing he might hurt them.

The patient has been on various medications at different doses over the years but has not been compliant due to his mother’s negligence in refilling prescriptions and missing follow-ups. She reported that he sleeps well on risperidone, has a good appetite, and has no decrease in energy at home. She denied any delusions or auditory hallucinations but mentioned that he is afraid of the dark, dislikes being alone in a room, and becomes distressed when the door is closed. The patient was in our ED, abandoned by his family for two months, as no one was willing to take him back due to his inability to control his suicidal thoughts. He was given appropriate medications and is currently under private child support with regular follow-up. A complaint of child neglect has been raised against his parent.

## Discussion

ASD encompasses a range of intricate developmental disorders marked by difficulties in social communication, limited interests, and repetitive behaviors. The extent of challenges varies greatly from person to person [1]. ASD affects approximately 2.3% of children aged 8 years and about 2.2% of adults in the United States [2]. Our patient, a 13-year-old boy with a long-standing diagnosis of ASD and ADHD, presents a severe case illustrating the intersection of these disorders with significant suicidal and homicidal ideations and behaviors. Individuals with ASD are at an increased risk for suicidal behaviors, including suicidal ideation, attempts, and death by suicide, compared to the general population​ [4]. Studies have shown that this increased risk is partly due to the overlap of genes associated with both ASD and suicidal behavior​ [4]. Weiner et al. presented a case of suicidal ideation in patients with ASD but no depression or other psychiatric illness. In this case, they stressed that ASD independently can be associated with the risk of suicide [6]. Social and communication impairments, common in individuals with ASD, have been previously identified as risk factors for suicide attempts​ [5]. This patient's significant difficulties in social interaction, combined with a history of aggressive behavior and isolation from peers and family members, likely contributed to his severe distress and suicidal tendencies. The case highlights the need for targeted interventions that address the unique social challenges faced by individuals with ASD to mitigate their risk of suicidal behaviors.

Our patient also has a history of ADHD, which further complicates his clinical picture. ADHD comorbid with ASD significantly increases the risk of suicidal behaviors. The odds of suicide attempts are notably higher in individuals with both ASD and ADHD, with one study reporting an odds ratio of 7.25 for suicide attempts in this population​ [3]. This comorbidity underscores the importance of careful monitoring and management of ADHD symptoms in patients with ASD to reduce the risk of suicide. Moreover, behavioral problems and lower socioeconomic status are identified risk factors for suicidality in ASD populations​ [7]. Our patient’s behavioral issues, including aggression toward his siblings and teachers, along with his family's social challenges and the presence of social stressors, likely exacerbated his suicidal behaviors. His mother's mental health issues and the family’s neglect further compounded his risk. The prevalence of suicidal ideation and attempts in individuals with ASD varies widely, with studies reporting ideation rates between 11% and 66% and attempt rates from 1% to 35%​ [8]. There are also studies that report cases in which autism was diagnosed only after the first hospitalization of the patient due to a suicide attempt [9]. This case aligns with the higher end of these estimates, reflecting the severe impact of untreated and poorly managed ASD and ADHD on mental health.

Finally, the systemic review by Balazs and Kereszteny highlights the positive association between ADHD and suicidality across all age groups and both sexes, emphasizing the need for comprehensive treatment plans that address all comorbid conditions [10]. The complexity of managing such cases requires a multidisciplinary approach, including regular follow-ups, medication compliance, and psychological support to prevent escalation to suicidal or homicidal actions.

One guiding principle is that suicide among autistic teenagers is preventable, and action by being vigilant and responsive can be taken by people involved in care with this age group, such as parents, teachers, and professionals. The current study is a single case and, therefore, lacks generalizability. Radiological and electroencephalogram findings were lost during follow-up, which is also another limitation of our study. The exploration of a single case provides further insight into similar cases in the future. To overcome these gaps, a meta-analysis should be performed to make more quantitative data and information available.

## Conclusions

This case underscores the critical need for early, consistent, and comprehensive interventions for individuals with ASD and ADHD. The interplay between these disorders and the associated risk of severe behavioral issues and suicidality necessitates a coordinated effort among healthcare providers, caregivers, and support systems to ensure the safety and well-being of affected individuals. The tragic outcomes observed in this patient’s history serve as a powerful reminder of the importance of adherence to treatment and the provision of robust support structures.
